# The G protein biased serotonin 5-HT2A receptor agonist lisuride exerts anti-depressant drug-like activities in mice

**DOI:** 10.3389/fmolb.2023.1233743

**Published:** 2023-10-10

**Authors:** Vladimir M. Pogorelov, Ramona M. Rodriguiz, Bryan L. Roth, William C. Wetsel

**Affiliations:** ^1^ Department of Psychiatry and Behavioral Sciences, Duke University Medical Center, Durham, NC, United States; ^2^ Mouse Behavioral and Neuroendocrine Analysis Core Facility, Duke University Medical Center, Durham, NC, United States; ^3^ Department of Pharmacology, University of North Carolina at Chapel Hill School of Medicine, Chapel Hill, NC, United States; ^4^ Center for Integrative Chemical Biology and Drug Discovery, Division of Chemical Biology and Medicinal Chemistry, Eshelman School of Pharmacy, National Institute of Mental Health Psychoactive Drug Screening Program, University of North Carolina at Chapel Hill School of Medicine, Chapel Hill, NC, United States; ^5^ Departments of Cell Biology and Neurobiology, Duke University Medical Center, Durham, NC, United States

**Keywords:** lisuride, *β*-arrestin, serotonin 2A receptor, mice, prepulse inhibition, head twitch, serotonin-syndrome

## Abstract

There is now evidence from multiple Phase II clinical trials that psychedelic drugs can exert long-lasting anxiolytic, anti-depressant, and anti-drug abuse (nicotine and ethanol) effects in patients. Despite these benefits, the hallucinogenic actions of these drugs at the serotonin 2A receptor (5-HT2AR) limit their clinical use in diverse settings. Activation of the 5-HT2AR can stimulate both G protein and *β*-arrestin (βArr) -mediated signaling. Lisuride is a G protein biased agonist at the 5-HT2AR and, unlike the structurally-related lysergic acid diethylamide (LSD), the drug does not typically produce hallucinations in normal subjects at routine doses. Here, we examined behavioral responses to lisuride, in wild-type (WT), βArr1-knockout (KO), and βArr2-KO mice. In the open field, lisuride reduced locomotor and rearing activities, but produced a U-shaped function for stereotypies in both βArr lines of mice. Locomotion was decreased overall in βArr1-KOs and βArr2-KOs relative to wild-type controls. Incidences of head twitches and retrograde walking to lisuride were low in all genotypes. Grooming was decreased in βArr1 mice, but was increased then decreased in βArr2 animals with lisuride. Serotonin syndrome-associated responses were present at all lisuride doses in WTs, but they were reduced especially in βArr2-KO mice. Prepulse inhibition (PPI) was unaffected in βArr2 mice, whereas 0.5 mg/kg lisuride disrupted PPI in βArr1 animals. The 5-HT2AR antagonist MDL100907 failed to restore PPI in βArr1 mice, whereas the dopamine D2/D3 antagonist raclopride normalized PPI in WTs but not in βArr1-KOs. Clozapine, SCH23390, and GR127935 restored PPI in both βArr1 genotypes. Using vesicular monoamine transporter 2 mice, lisuride reduced immobility times in tail suspension and promoted a preference for sucrose that lasted up to 2 days. Together, it appears βArr1 and βArr2 play minor roles in lisuride’s actions on many behaviors, while this drug exerts anti-depressant drug-like responses without hallucinogenic-like activities.

## 1 Introduction

Lisuride was first synthesized in 1960 as an analog of methysergide (Zikán and Semonský, 1960) and, as an ergoline derivative, it has a chemical structure similar D-lysergic acid diethylamide (LSD). Both lisuride and LSD bind with high affinities to serotonin (5-HT) 2A receptors (5-HT2AR) and signal through Gαq by activation of phospholipase C leading to production of inositol phosphates and diacylglycerol with release of intracellular Ca^2+^ *in vitro* (Hoyer et al., 1994; Egan et al., 1998; Kurrasch-Orbaugh et al., 2003; Cussac et al., 2008). Additionally, 5-HT2AR agonists can stimulate phospholipase A_2_ and mediate release of arachidonic acid (Felder et al., 1990; Kurrasch-Orbaugh et al., 2003). While lisuride and LSD are partial 5-HT2AR agonists (Egan et al., 1998; Kurrasch-Orbaugh et al., 2003; Wacker et al., 2013; Zhang et al., 2022), they bind also dopaminergic, adrenergic, and other serotonergic receptors (Piercey et al., 1996; Egan et al., 1998; Marona-Lewicka et al., 2002; Millan et al., 2002; Kroeze et al., 2015). Despite these similarities, LSD possesses hallucinogenic activity in humans at doses as low as 20 µg (Greiner et al., 1958), while lisuride is devoid of these psychedelic effects up to 600 µg in normal subjects (Herrmann et al., 1977).

Although lisuride is reported to produce hallucinations in some patients with Parkinson’s disease (Schachter et al., 1979; Parkes et al., 1981; LeWitt et al., 1982; Vaamonde et al., 1991), these effects may be attributable to dysregulation of dopaminergic and other neurotransmitter systems in this disease. Nevertheless, the lack of hallucinations with lisuride in normal subjects seems surprising since agonism at the 5-HT2AR mediates the hallucinogenic actions of psychedelics (Glennon et al., 1983; Schreiber et al., 1995; Keiser et al., 2009) and because antagonistic actions by atypical antipsychotic drugs diminish the psychosis associated both with schizophrenia and Parkinson’s psychosis (Roth et al., 2004; Meltzer and Roth, 2013). To date, lisuride has been used in humans to treat migraine and cluster headache, parkinsonism, and hyperprolactinemia (Herrmann et al., 1977; Verde et al., 1980; McDonald and Horowski, 1983; Raffaelli et al., 1983). By comparison, LSD has potential efficacy in treating cluster headache, anxiety and depression in life-threatening situations when combined with psychotherapy, and it may be useful in studying consciousness and treating substance abuse (Sewell et al., 2006; Gasser et al., 2015; Bogenschutz and Johnson, 2016; Carhart-Harris et al., 2016; Griffiths et al., 2016; Goodwin et al., 2023; Holze et al., 2023). Hence, the therapeutic profiles of these drugs are different.

In rodents the head twitch response (HTR) to psychedelics is mediated by 5-HT2AR activation (Glennon et al., 1984; González-Maeso et al., 2007; Keiser et al., 2009) and is used frequently to determine the potential psychedelic actions of drugs. However, several non-hallucinogenic drugs including quipazine, 5-hydroxytryptophan, ergometrine, cannabinoid CB1 antagonists, rolipram, fenfluramine, and other drugs also induce HTRs (Corne and Pickering, 1967; Malick et al., 1977; Wachtel, 1983; Darmani, 1998; Darmani and Pandya, 2000), although to our knowledge there are no classical psychedelic drugs that do not induce HTRs. Thus, while there are many false positives for the HTR, we are unaware of false negative hallucinogens. In contrast to LSD and other psychedelics, lisuride does not produce HTRs in rats (Gerber et al., 1985) or mice (González-Maeso et al., 2007; Halberstadt and Geyer, 2013). Besides HTRs, another distinction between lisuride and LSD is their differential agonist profile at the 5-HT2AR. While both ligands bind this receptor, the residues in the binding pocket bound by these ligands produce slightly different receptor conformations and may show differential responses through G protein- and *β*-arrestin (βArr) -mediated signaling (Kim et al., 2020; Cao et al., 2022). Parenthetically, the ability of ligands to stimulate or inhibit different pathways from the same receptor is termed functional selectivity or biased signaling (Roth and Chuang, 1987; Kenakin, 1995). Recently, LSD has been shown to be βArr biased at the 5-HT2AR *in vitro* (Wacker et al., 2017; Cao et al., 2022) and *in vivo* (Rodriguiz et al., 2021).

In present study we used βArr1 and βArr2 mice to determine if lisuride could exert differential effects on motor performance, PPI, and various other behaviors—including HTRs--in the presence and absence of βArrs. As there is emerging evidence that psychedelics and 5-HT2AR agonists have anti-depressant actions in humans (Carhart-Harris et al., 2016; Griffiths et al., 2016; McClure-Begley and Roth, 2022; Goodwin et al., 2023), we tested if lisuride had similar actions in the vesicular monoamine transporter 2 (VMAT2) heterozygous (HET) mice (see Fukui et al., 2007). Note, these mutants were selected for study because they phenocopy the hypertensive patients that were treated with the VMAT inhibitor, reserpine, who experienced depression without anxiety (Freis, 1954), and because these findings were used initially as a basis for the monoamine hypothesis of depression (Schildkraut, 1965; Maes et al., 1994).

## 2 Results

### 2.1 Lisuride reduces motor activities in βArr1 and βArr2 mice

**FIGURE 1:**
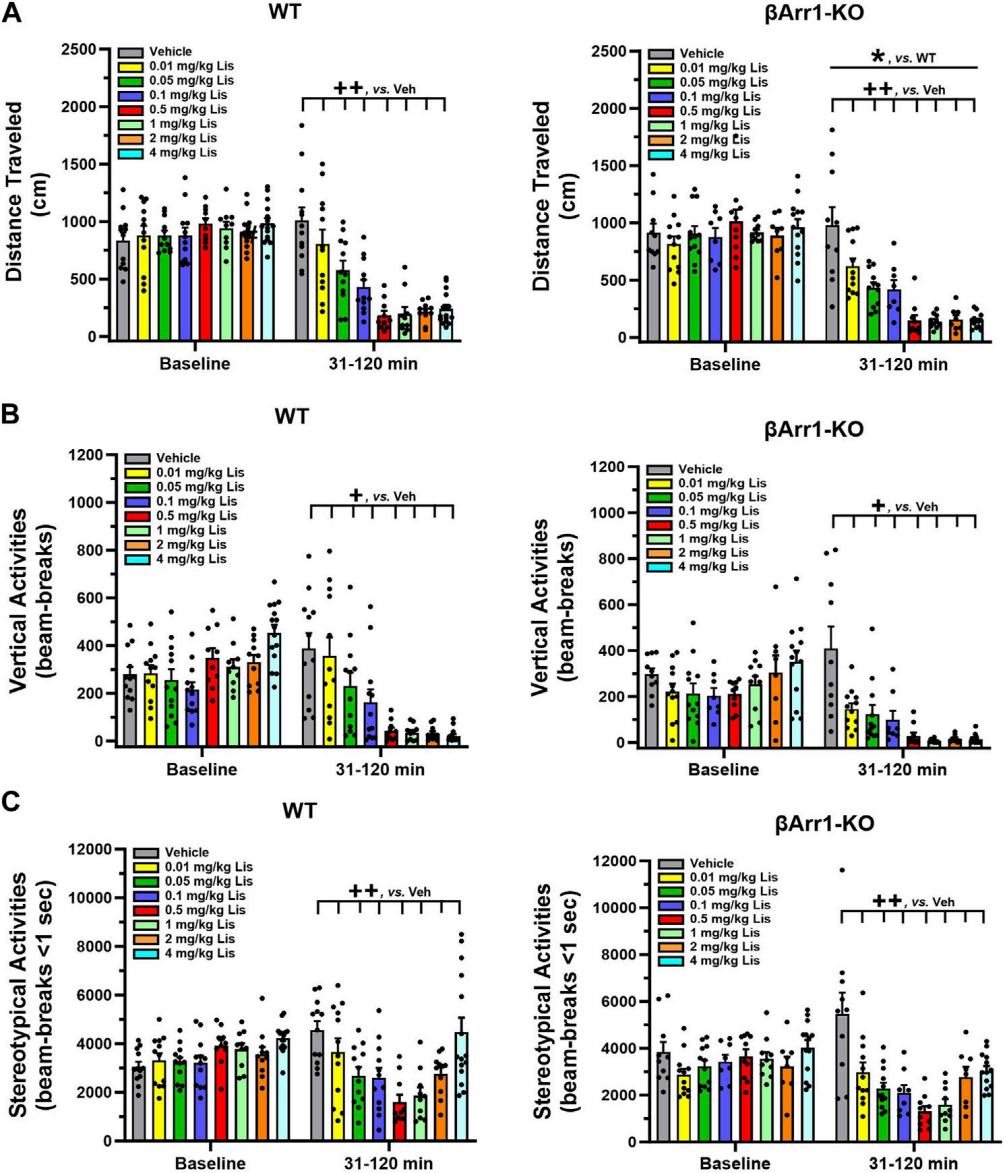
Effects of lisuride on cumulative motor activities in *β*-arrestin 1 mice. Baseline activities were monitored from 0 to 30 min, animals were given vehicle (Veh) or various doses of lisuride, and returned to the open field for 90 min. **(A)** Cumulative distance traveled. Baseline: no significant effects. Post-injection: two-way ANOVA for genotype [F (1,161) = 4.050, *p* = 0.046] and treatment [F (7,161) = 33.270, *p* < 0.001]. **(B)** Vertical counts (rearing). Baseline: two-way ANOVA for genotype [F (1,161) = 7.636, *p* = 0.006] and treatment [F (7,161) = 5.384, *p* < 0.001]. Post-administration: two-way ANCOVA for treatment [F (7,160) = 27.412, *p* < 0.001]. **(C)** Stereotypies. Baseline: two-way ANOVA for treatment [F (7,161) = 3.298, *p* = 0.003]. Following injection: two-way ANCOVA for treatment [F (7,160) = 14.609, *p* < 0.001]. Data presented as means ±SEMs; n = 8–15 mice/genotype/treatment; *p < 0.05, WT vs. KO; ^+^ *p*< 0.05, ^++^ *p*< 0.01, vs. Vehicle.

When cumulative locomotor activities were examined at baseline in βArr1 animals, no significant genotype or treatment effects were found (Figure 1A). Following drug administration, overall cumulative locomotor activities were lower in βArr1-KO than WT mice (*p* = 0.046). With lisuride, locomotion in both genotypes decreased in a dose-dependent fashion from 0.01 to 0.5 mg/kg lisuride relative to vehicle (*p*-values≤0.005) and remained flat.

An examination of cumulative rearing found basal responses between βArr1 genotypes (*p* = 0.006) and their assigned treatment groups to be different (*p* < 0.001) (Figure 1B). To control for these conditions, post-administration cumulative rearing activities were subjected to analysis of covariance (ANCOVA). No significant genotype effects were detected; however, the initial suppression in rearing was visually more apparent in βArr1-KO than WT animals. Rearing activities in both genotypes decreased dose-dependently from vehicle to 0.05 mg/kg lisuride (*p*-values≤0.036) and remained low to the 4 mg/kg dose.

Basal cumulative stereotypies were different among βArr1 mice prior to treatment (*p* = 0.003) (Figure 1C). While no genotype effects at post-injection were noted with ANCOVA, cumulative stereotypies assumed a U-shaped function for dose with a decline from vehicle through 0.01–0.5 mg/kg lisuride (*p*-values≤0.009) and then these activities increased with 4 mg/kg lisuride but were still below that of vehicle (*p* < 0.008).

**FIGURE 2:**
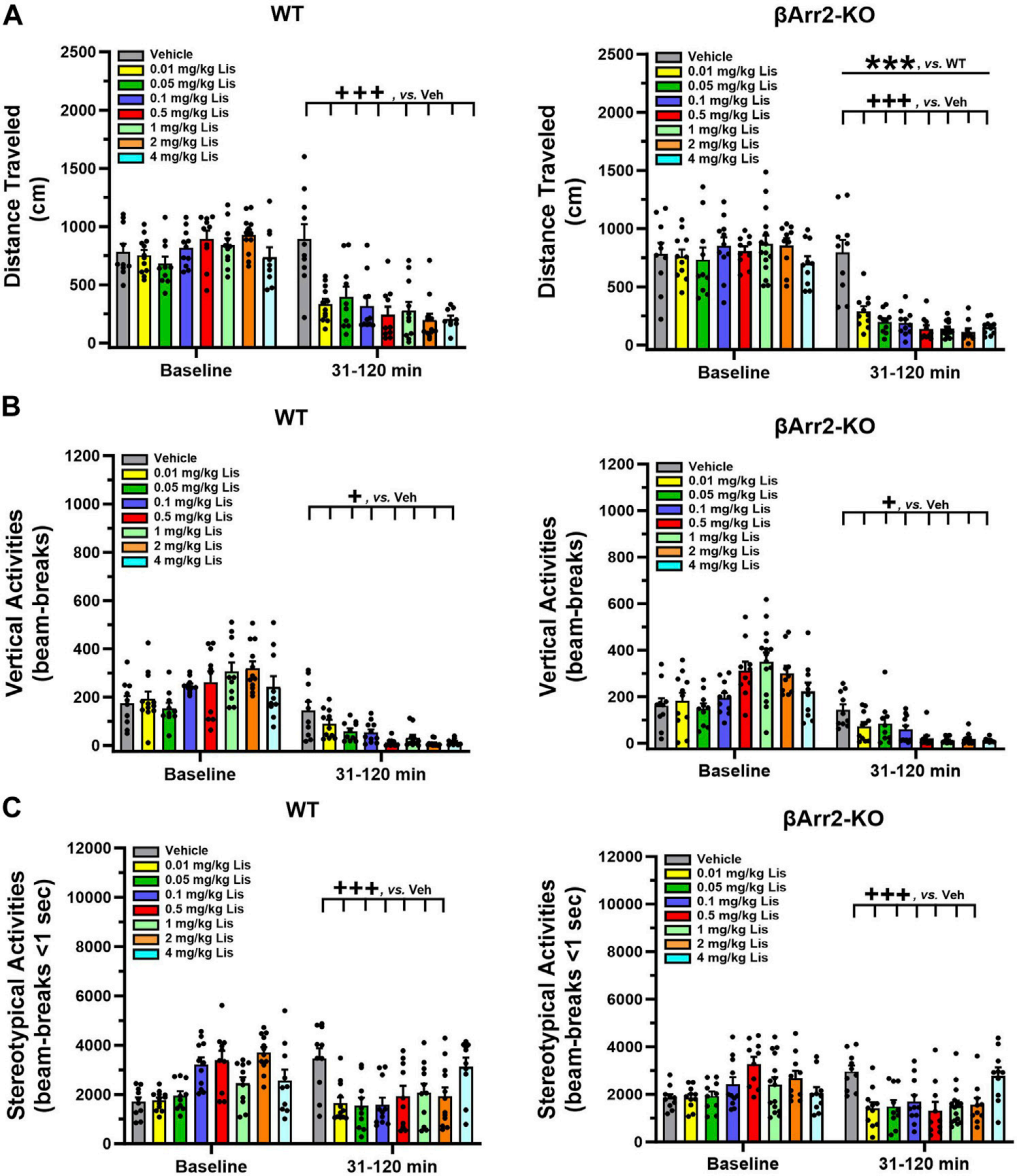
Effects of lisuride on cumulative motor activities in *β*-arrestin 2 mice. The procedure is identical to Figure 1. **(A)** Cumulative distance traveled. Baseline: no significant effects. Post-injection: two-way ANOVA for genotype [F (1,157) = 11.710, *p* < 0.001] and treatment [F (7,157) = 25.825, *p* < 0.001]. **(B)** Vertical counts (rearing). Baseline: two-way ANOVA for treatment [F (7,157) = 8.162, *p* < 0.001]. Post-administration: two-way ANCOVA for treatment [F (7,156) = 18.319, *p* < 0.001]. **(C)** Stereotypical activities. Baseline: two-way ANOVA for genotype [F (1,157) = 4.205, *p* = 0.042] and treatment [F (7,157) = 10.009, *p* < 0.001]. Following injection: two-way ANCOVA for treatment [F (7,156) = 8.485, *p* < 0.001]. Data presented as means ±SEMs; n = 10–15 mice/genotype/treatment. ***p < 0.001, WT vs. KO; ^+^ *p*< 0.05, ^+++^ *p*< 0.001, vs. Vehicle.

Motor activities were evaluated also in βArr2 mice. No genotype or treatment effects were discerned for baseline cumulative locomotion (Figure 2A). Nonetheless, post-administration a lisuride-induced depression in activity was more robust in βArr2-KO than WT mice (*p* < 0.001). All doses of lisuride suppressed locomotion compared to vehicle (*p*-values<0.001).

An assessment of cumulative rearing in βArr2 animals revealed basal responses to be different across treatment assignments (*p* < 0.001) (Figure 2B). The ANCOVA determined post-administration cumulative rearing was depressed from vehicle though all lisuride doses (*p*-values≤0.028).

Basal cumulative stereotypies were differentiated by genotype (*p* = 0.042) and treatment assignment (*p* < 0.001) (Figure 2C). Following injection of vehicle or different doses of lisuride, ANCOVA discerned a trend for a genotype effect (*p* = 0.056) where responses were lower overall in βArr2-KO than WT mice. Treatment effects were more dramatic and were U-shaped. Here, the numbers of cumulative stereotypies declined from vehicle to 0.01 lisuride (*p* < 0.001), responses were low to 2 mg/kg (*p*-values≤0.001), then ascended with 4 mg/kg lisuride to the vehicle control.

Taken together, these findings show that in both βArr1 and βArr2 mice lisuride reduces cumulative locomotor, rearing, and stereotypical activities; however, these latter activities assume a biphasic U-shaped function with lisuride.

### 2.2 Lisuride produces serotonin syndrome-associated behaviors

**FIGURE 3:**
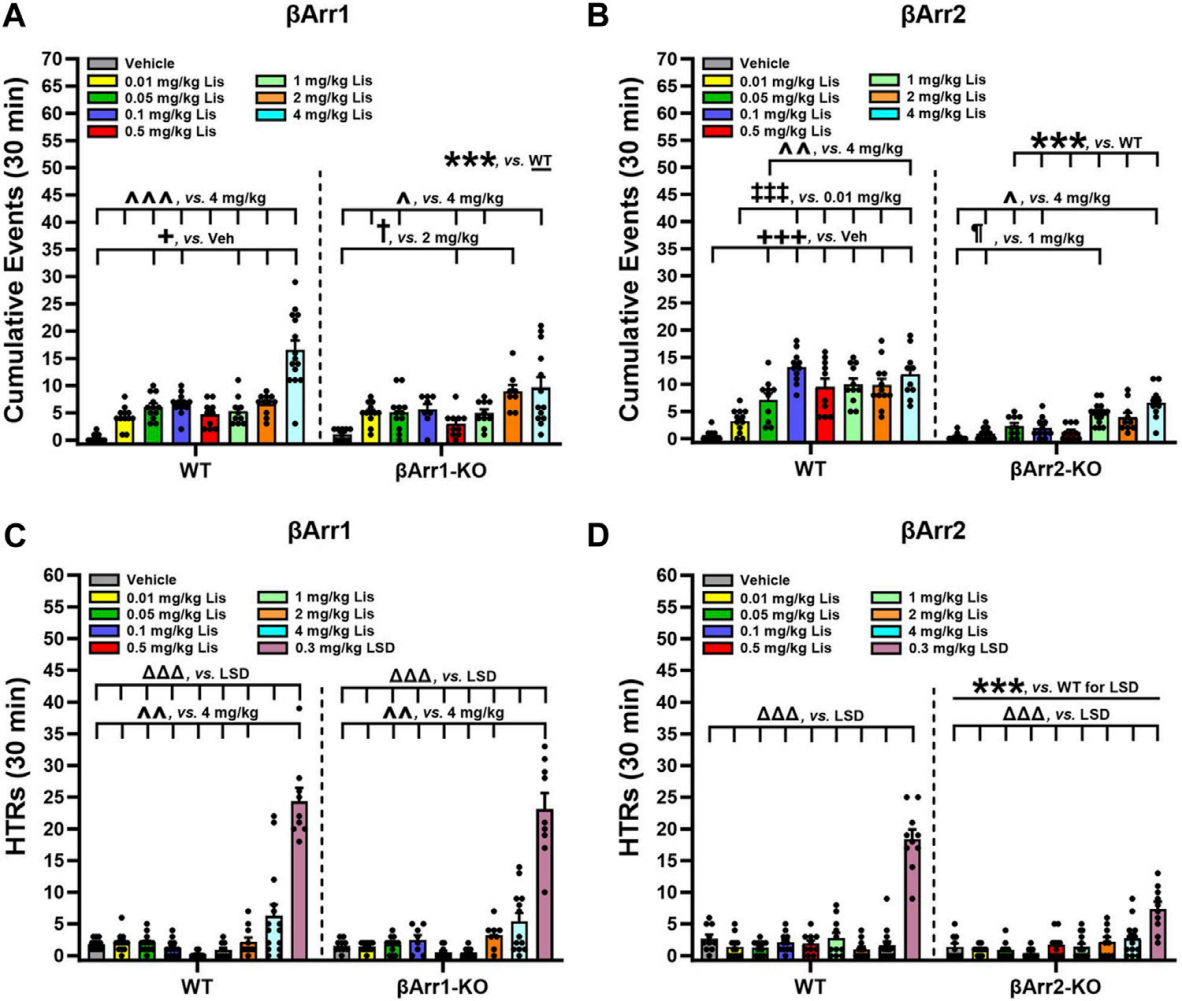
Serotonin syndrome-associated behaviors and head twitch responses with lisuride in *β*-arrestin 1 and *β*-arrestin 2 mice. Responses were scored over the initial 30-min post-injection period. **(A)** Lisuride dose-response induced 5-HT syndrome-associated behaviors in *β*-arrestin 1 mice. Two-way ANOVA for treatment [F (7,161) = 26.264, *p* < 0.001] and genotype by treatment interaction [F (7,161) = 3.918, *p* < 0.001]; n = 8–15 mice/genotype/treatment. **(B)** Lisuride dose-response induced 5-HT syndrome-associated behaviors in *β*-arrestin 2 mice. Two-way ANOVA for genotype [F (1,157) = 153.263, *p* < 0.001], treatment [F (7,157) = 22.422, *p* < 0.001], and genotype by treatment interaction [F (7,157) = 7.193, *p* < 0.001]; n = 10–15 mice/genotype/treatment. **(C)** Head twitch responses in βArr1 mice given different doses of lisuride and 0.3 mg/kg LSD. Two-way ANOVA for treatment [F (8,177) = 89.256, *p* < 0.001]; n = 8–15 βArr1 mice/genotype/treatment. **(D)** Head twitch responses in βArr2 mice receiving the same treatments as panel C. Two-way ANOVA for genotype [F (1,175) = 23.388, *p* < 0.001], treatment [F (8,175) = 57.323, *p* < 0.001], and genotype by treatment interaction [F (8,175) = 13.667, *p* < 0.001]; n = 10–15 βArr2 mice/genotype/treatment. The data are presented as means ±SEMs. ***p < 0.001, WT vs. KO for Lisuride or 0.3 mg/kg LSD; ^+^ *p*< 0.05, ^+++^ *p*< 0.001, vs. Vehicle; ^‡‡‡^ *p* < 0.001, vs. 0.01 mg/kg Lisuride; ^¶^ *p* < 0.05, vs. 1 mg/kg Lisuride; ^†^ *p* < 0.05, vs. 2 mg/kg Lisuride; ^^^ *p* < 0.05, ^^^^ *p* < 0.01, ^^^^^ *p* < 0.001, vs. 4 mg/kg Lisuride; ^ΔΔΔ^p<0.001, vs. LSD.

To determine which behaviors were expressed as stereotypies, responses were scored over 30 min immediately following lisuride administration. We identified many behaviors associated with 5-HT syndrome and they consisted of hunched posture, repetitive paw kneading, rigid posture, flat posture, Staub tail, and tremor or body shaking (see Haberzettl et al., 2013). HTRs and retrograde walking may be included also and these are presented later. When cumulative responses listed initially above were tabulated across doses, 4 mg/kg lisuride elicited more responses in WT than βArr1-KO mice (*p* < 0.001) (Figure 3A; Supplementary Figure S1). Within WT mice 5-HT syndrome-associated responses were expressed at higher levels with 0.05, 0.1, 1, 2 and 4 mg/kg lisuride than vehicle (*p*-values≤0.034). Importantly, the numbers of responses with 4 mg/kg lisuride were enhanced over all other lisuride doses (*p*-values<0.001). In βArr1-KO mice the incidences of 5-HT syndrome-associated responses were highest also with 4 mg/kg relative to vehicle and 0.01, 0.05, 0.5, and 1 mg/kg lisuride (*p*-values≤0.041); responses to 2 mg/kg lisuride were increased over vehicle and the 0.5 mg/kg dose (*p*-values≤0.013).

Genotype differences were robust in βArr2 mice, where all doses but 0.01 mg/kg lisuride stimulated the expression of more 5-HT syndrome-associated responses in WT than βArr2-KO animals (*p*-values≤0.001) (Figure 3B; Supplementary Figure S2). Within WT mice, 0.05–4 mg/kg lisuride stimulated more responses than vehicle (*p*-values<0.001) and 0.1–4 mg/kg lisuride produced more responses than the 0.01 mg/kg dose (*p*-values<0.001). In addition, responses to 0.1 and 4 mg/kg lisuride were higher than for the 0.05 mg/kg dose (*p*-values≤0.009). In βArr2-KO animals, responses to the 1 and 4 mg/kg doses were enhanced over vehicle and 0.01 mg/kg lisuride (*p*-values≤0.033). The 4 mg/kg lisuride-induced responses were higher also than to 0.01 and 0.1 mg/kg lisuride (*p*-values≤0.028). Collectively, these results show that 5-HT syndrome-associated responses are elicited at much higher rates in WT than βArr2-KO mice to 0.05–4 mg/kg lisuride, while only the highest lisuride dose augments more responses in WTs than βArr1-KO animals.

### 2.3 Lisuride effects on additional stereotyped behaviors in βArr1 and βArr2 mice

When HTRs were examined, both WT and βArr1-KO mice responded similarly to lisuride (Figure 3C). Here 4 mg/kg lisuride stimulated more HTRs than all other doses except 2 mg/kg (*p*-values≤0.004). Even with this high 4 mg/kg dose less than 7 HTRs were identified over the 30-min post-injection period, whereas LSD stimulated high levels of HTRs relative to all other treatments (*p*-values<0.001). In βArr2 animals, the numbers of HTRs barely exceeded 3 with 4 mg/kg lisuride and this number was not significantly different from vehicle (Figure 3D). By contrast, LSD stimulated more HTRs in WT relative to βArr2-KO animals (*p* < 0.001). Thus, with lisuride HTRs are exceedingly low in both βArr lines, while LSD stimulates more HTRs in βArr1-KO and WTs than βArr2-KO mice.

For self-grooming, no genotype effects were detected in either βArr strain (Supplementary Figure S3A,B). Compared to vehicle, time spent grooming in βArr1 mice decreased with 0.05–1 and 4 mg/kg lisuride (*p*-values ≤0.030) (Supplementary Figure S3A). By contrast, in βArr2 animals lisuride produced an inverted U-shaped function (Supplementary Figure S3B). From a low at 0.01 mg/kg lisuride, grooming increased with the 0.05 mg/kg dose (*p* = 0.021) and it remained high to 0.5 mg/kg lisuride, then declined with the highest dose (*p* = 0.006).

For retrograde walking, no genotype effects were found with lisuride (Supplementary Figure S3C). Retrograde walking assumed a biphasic U-shaped curve across doses. Lisuride decreased this behavior from 0.01 mg/kg lisuride to the 0.05 and 0.5 mg/kg doses (*p*-values<0.001), whereas 2 and 4 mg/kg lisuride augmented the response from the 0.5 and 1 mg/kg doses (*p*-values≤0.021). With βArr2 mice, a biphasic dose-response curve was found (Supplementary Figure S3D). Declines from vehicle were seen with the 0.1 mg/kg dose (*p* = 0.025), while retrograde events were increased from this nadir with 4 mg/kg lisuride.

Collectively, only treatment effects with lisuride are obtained for HTRs, grooming, and retrograde walking. Overall HTRs are higher in βArr1 than βArr2 mice, but they are very low compared to LSD. The duration of grooming is decreased in βArr1 mice, but is enhanced in βArr2s except at 4 mg/kg lisuride. By contrast, the incidences of retrograde walking are represented by a U-shaped function in both mouse strains.

### 2.4 Prepulse inhibition is reduced by lisuride in βArr1 but not βArr2 mice

**FIGURE 4:**
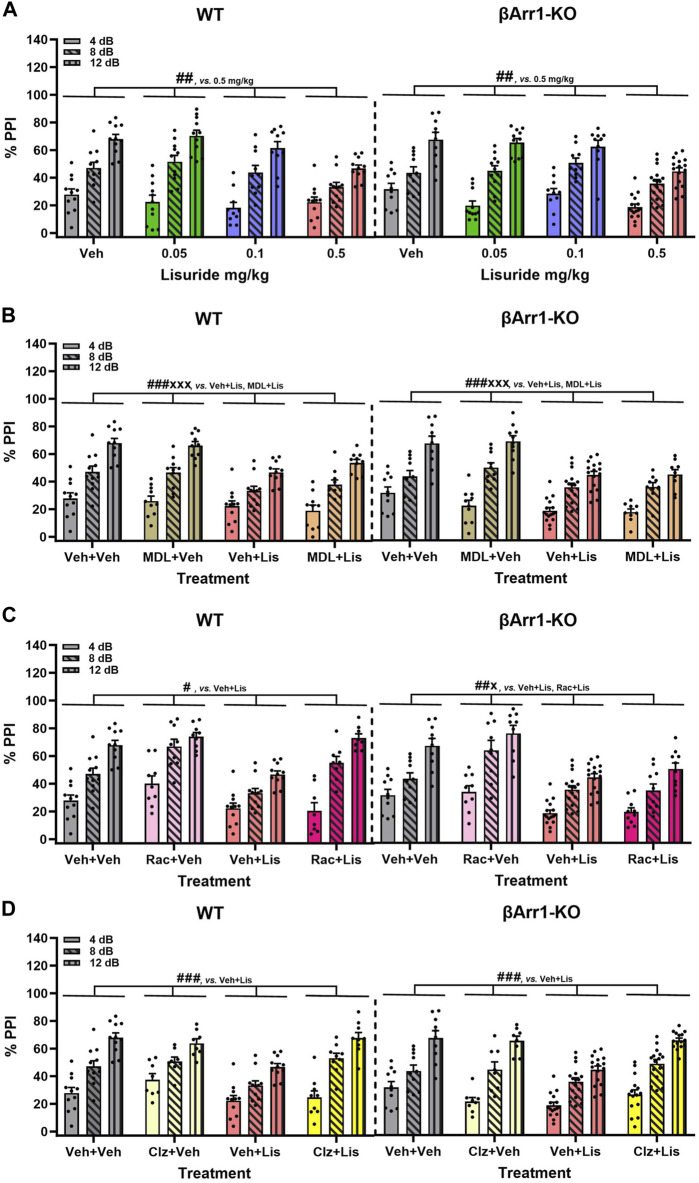
Effects of lisuride on prepulse inhibition in *β*-arrestin 1 mice. Animals were administered vehicle, 0.5 mg/kg MDL100907, 3 mg/kg raclopride, or 0.5 mg/kg clozapine before giving vehicle or lisuride and tested. **(A)** PPI in βArr 1 mice receiving vehicle or different lisuride doses. RMANOVA for PPI [F (2,164) = 406.059, *p* < 0.001], PPI by treatment interaction [F (6,164) = 7.644, *p* < 0.001], and treatment [F (3,82) = 8.814, *p* < 0.001]; n = 10–16 βArr1 mice/genotype/treatment. **(B)** PPI in βArr1 mice treated with vehicle, 0.5 mg/kg MDL100907, 0.5 mg/kg lisuride, or MDL100907 plus lisuride. RMANOVA for PPI [F (2,158) = 368.350, *p* < 0.001], PPI by treatment interaction [F (6,158) = 6.158, *p* < 0.001], and treatment [F (3,79) = 14.448, *p* < 0.001]; n = 9–16 βArr1 mice/genotype/treatment. **(C)** PPI in βArr1 mice given vehicle, 3 mg/kg raclopride, 0.5 mg/kg lisuride, or raclopride plus lisuride. RMANOVA for PPI [F (2,152) = 450.960, *p* < 0.001], PPI by treatment interaction [F (6,152) = 8.922, *p* < 0.001], PPI by genotype by treatment interaction [F (6,152) = 5.510, *p* < 0.001], and treatment [F (3,76) = 22.609, *p* < 0.001]. Deconstruction of the 3-way interaction with univariate tests for WT [F (3,76) = 9.933, *p* < 0.001] and βArr1-KO mice [F (3,76) = 14.798, *p* < 0.001]; n = 8–16 βArr1 mice/genotype/treatment. **(D)** PPI in βArr1 mice administered vehicle, 0.5 mg/kg clozapine, 0.5 mg/kg lisuride, or clozapine plus lisuride. RMANOVA for PPI [F (2,160) = 339.837, *p* < 0.001], PPI by treatment interaction [F (6,160) = 5.079, *p* < 0.001], PPI by genotype by treatment interaction [F (6,160) = 2.191, *p* = 0.046], and treatment [F (3,80) = 13.641, *p* < 0.001]; n = 8–16 βArr1 mice/genotype/treatment. ^#^ *p* < 0.05, ^##^ *p* < 0.01, ^###^ *p* < 0.001, vs. Vehicle + Lisuride; ^x^p < 0.05, ^xxx^p<0.001, Antagonist + Lisuride.

LSD- and certain other psychedelic-induced states share many similarities with early acute phases of psychosis (Geyer and Vollenweider, 2008). PPI is abnormal in schizophrenia (Braff et al., 2001) and LSD disrupts PPI in rats (Ouagazzal et al., 2001; Halberstadt and Geyer, 2010; Páleníček et al., 2010) and mice (Rodriguiz et al., 2021). Since the chemical structure of lisuride is similar to LSD, effects of lisuride on PPI were analyzed in both βArr strains. No genotype effects were detected with βArr1 mice (Figure 4A). Nonetheless, 0.5 mg/kg lisuride reduced PPI overall relative to all treatment-groups (*p*-values ≤0.007). Hence, subsequent experiments were conducted with 0.5 mg/kg lisuride and different receptor antagonists to determine if they could normalize PPI.

The 5-HTR2A antagonist, MDL100907, was tested first in βArr1 animals (Figure 4B). No genotype effects were identified. Although lisuride reduced PPI relative to vehicle and 0.5 mg/kg MDL100907 (*p*-values<0.001), the antagonist failed to normalize PPI (*p* < 0.001).

The dopamine D2/D3 (D2/D3) antagonist, raclopride, was evaluated next. Here, the lisuride disruption of PPI in WT mice was normalized with 3 mg/kg raclopride (*p* = 0.018) (Figure 4C). In βArr1-KO mice, raclopride failed to rescue PPI to the vehicle and raclopride control levels (*p*-values≤0.045).

**FIGURE 5:**
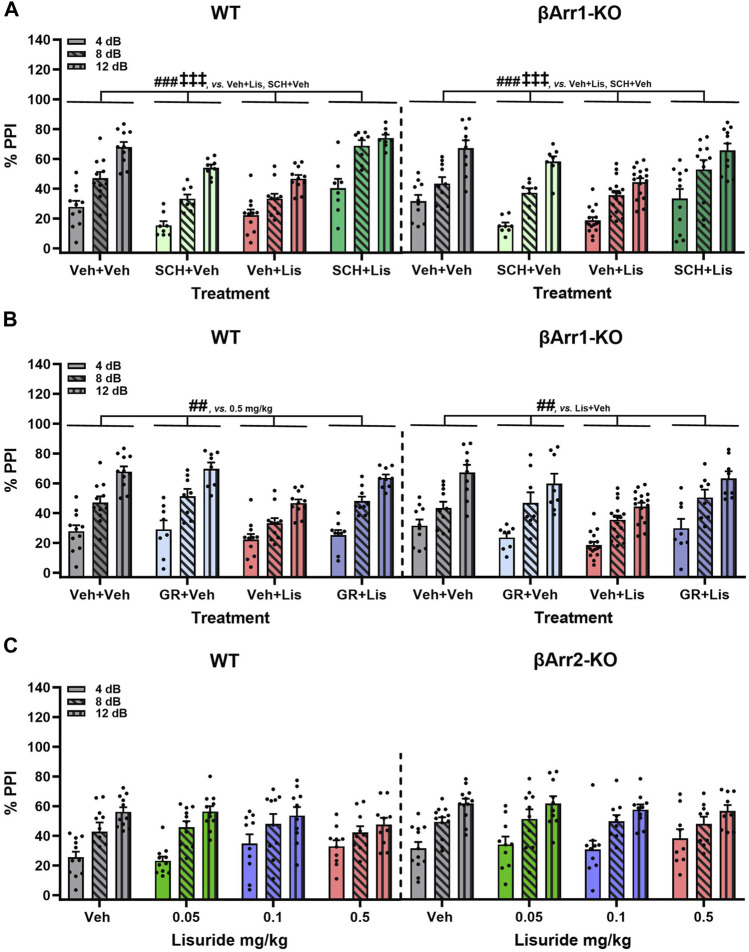
Effects of lisuride on prepulse inhibition in *β*-arrestin 1 and *β*-arrestin 2 mice. *β*-Arrestin 1 animals were administered vehicle, SCH23390, or GR127935 before giving vehicle or lisuride and tested. *β*-Arrestin 2 mice were injected with vehicle or different doses of lisuride and tested. **(A)** PPI in βArr1 animals given vehicle, 0.1 mg/kg SCH23390, 0.5 mg/kg lisuride, or SCH23390 plus lisuride. RMANOVA for PPI [F (2,150) = 334.367, *p* < 0.001], PPI by treatment interaction [F (6,150) = 5.705, *p* < 0.001], and treatment [F (3,75) = 19.741, *p* < 0.001]; n = 8–16 βArr1 mice/genotype/treatment. **(B)** PPI in βArr1 animals injected with vehicle, 2 mg/kg GR127935, 0.5 mg/kg lisuride, or GR127935 plus lisuride. RMANOVA for PPI [F (2,146) = 345,361, *p* < 0.001], PPI by treatment interaction [F (6,146) = 4.542, *p* < 0.001], and treatment [F (3,73) = 9.015, *p* < 0.001]; n = 8–16 βArr1 mice/genotype/treatment. **(C)** PPI in βArr 2 animals given vehicle or different doses of lisuride. RMANOVA for PPI [F (2,150) = 132.746, *p* < 0.001] and PPI by treatment interaction [F (6,150) = 2.485, *p* = 0.025]; n = 9–12 βArr2 mice/treatment. Data presented as means ±SEMs. ^‡‡‡^ *p* < 0.001, vs. Antagonist + Vehicle; ^##^ *p* < 0.01, ^###^ *p* < 0.001, vs. Vehicle + Lisuride.

Clozapine--another atypical anti-psychotic drug--with a broader spectrum of receptor targets—was tested (Figure 4D). In WT and βArr1-KO mice clozapine rescued PPI (*p*-values≤0.001). As lisuride binds to dopaminergic, adrenergic, and various serotonergic receptors (Piercey et al., 1996; Egan et al., 1998; Marona-Lewicka et al., 2002; Millan et al., 2002; Kroeze et al., 2015), additional antagonists were tested. Two receptors affecting PPI are D1R and 5-HTR1B/D (Ralph and Caine, 2005; Thompson and Dulawa, 2019). No genotype effects were noted with D1 (SCH23390) or 5-HTR1B/D (GR127935) antagonists (Figures 5A, B). The lisuride-induced disruption of PPI was restored in both βArr1 genotypes with 0.1 mg/kg SCH23390 (*p* < 0.001) (Figure 5A) and 2 mg/kg GR127935 (*p* = 0.001) (Figure 5B). In contrast to βArr1 mice, no genotype or treatment effects in PPI were observed when WT and βArr2-KO animals were given 0.05, 0.1, or 0.5 mg/kg lisuride (Figure 5C).

Besides PPI, null and startle activities were examined in both βArr1 and βArr2 mice where some genotype and treatment effects were noted (Supplementary Figures S4, S5). Nevertheless, most null responses were less than 10% of startle activities with all treatments.

In summary, PPI was disrupted in βArr1 mice given 0.5 mg/kg lisuride and MDL100907 was unable to reverse this effect. Raclopride normalized PPI but only in WT mice; PPI in βArr1-KOs remained abnormal. Clozapine, SCH23390, and GR127935 restored PPI in both WT and βArr1-KO mice. In βArr2 mice, PPI was retained with all lisuride doses.

### 2.5 Lisuride exerts anti-depressant drug-like actions in VMAT2 heterozygotes

WT and VMAT2-HET mice were used to assess anti-depressant drug-like actions of lisuride. Parenthetically Fukui and colleagues (2007) reported adult VMAT2-HETs were hypoactive, displayed anhedonia-like responses, and showed enhanced immobility in tail suspension and forced swim that were normalized with tricyclic anti-depressants, selective 5-HT and norepinephrine transporter inhibitors, and bupropion. These mutants were more responsive to stress and displayed enhanced learned helplessness compared to WTs.

**FIGURE 6:**
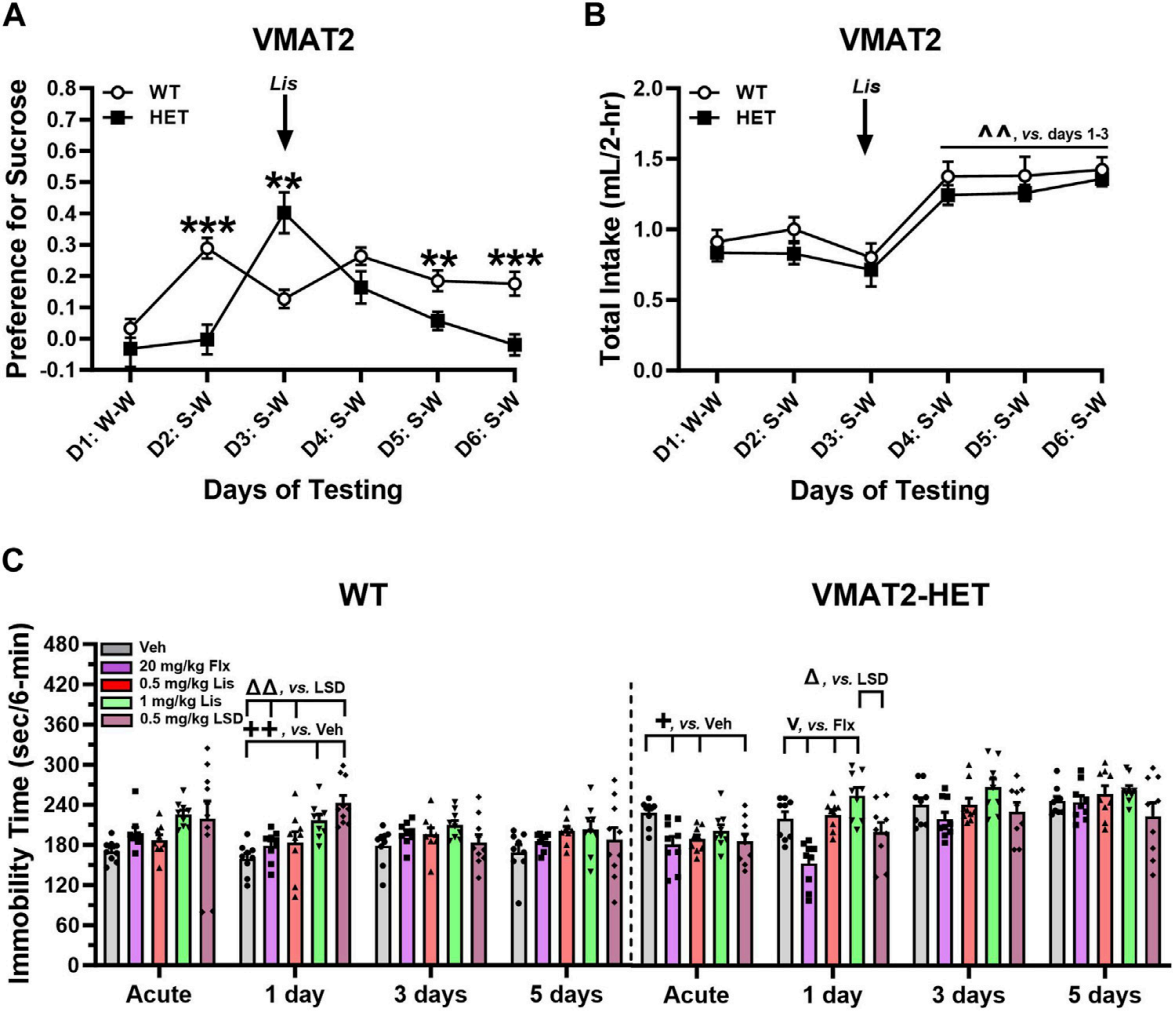
Effects of lisuride in tests of depressive-like behaviors in VMAT2 mice. In a two-bottle test, mice were presented with a water-water (W–W) pairing on day 1 and sucrose-water (S–W) pairings on days (D) 2–6; effects of vehicle (Veh) and lisuride (Lis) were examined. **(A)** Sucrose preference in VMAT2 mice. RMANOVA for day [F (5,110) = 8.939, *p* < 0.001], day by genotype interaction [F (5,110) = 9.414, *p* < 0.001], and genotype [F (1,22) = 7.863, *p* = 0.010]. **(B)** Total fluid intake. RMANOVA for day [F (5,110) = 21.585, *p* < 0.001]; n = 10–14 mice/genotype/treatment. **p < 0.01, ***p < 0.001, WT vs. VMAT2-HET; ^ ^^*p* < 0.01, vs. Days 1–3. **(C)** Tail suspension (6 min test). RMANOVA for immobility in WT mice: day by treatment interaction [F (12,123) = 2.758, *p* = 0.002] and treatment [F (4,41) = 4.591, *p* = 0.004]. RMANOVA for immobility in VMAT2-HET mice: day [F (3,120) = 26.715, *p* < 0.001], day by treatment interaction [F (12,120) = 2.901, *p* = 0.001], and treatment [F (4,40) = 8.027, *p* < 0.001]; n = 9–10 mice/genotype//treatment. All data presented as means ±SEMs. ^+^ *p*< 0.05, ^++^ *p*< 0.01, vs. Vehicle; ^v^p < 0.05, vs. Fluoxetine; ^Δ^p<0.05, ^ΔΔ^p<0.01, vs. LSD.

WT and VMAT2-HET mice were tested on day 1 with water-water (W-W) pairings where they showed no bottle or side-preference (Figure 6A). On day 2 mice were given vehicle and presented with a 0.5% sucrose-water (S-W) pairing. WTs had a strong preference for sucrose compared to mutants (*p* < 0.001), whose preference was similar to day 1 W-W. Following acute administration of 0.5 mg/kg lisuride on day 3, the VMAT2-HETs’ sucrose preference exceeded WTs (*p* = 0.003) and it was significantly higher from their selections on days 1 and 2 (*p*-values<0.001). On day 4, the mutants’ preference was similar to WT mice. However, by days 5 and 6 sucrose preference in VMAT2-HET mice fell from WT levels (*p*-values≤0.010) where their preference was similar to day 1 W-W. One reason why sucrose preference could vary across days in mutants might be due to differences in intake. However, no genotype differences were found across days (Figure 6B). Nonetheless, total intake significantly increased in all mice from days 1–3 to days 4–6 (*p*-values≤0.002). Collectively, VMAT2-HET mice fail to show a sucrose preference until they are given lisuride. This preference persists for 2 days and declines thereafter, whereas sucrose preference is maintained in WTs regardless of lisuride treatment. Importantly, these differences in sucrose preference cannot be attributed to differential fluid consumption between genotypes.

In tail suspension (6 min), VMAT2 mice were treated acutely with vehicle, 20 mg/kg fluoxetine, or 0.5 or 1 mg/kg lisuride and tested 30 min later. With 0.5 mg/kg LSD, mice were tested acutely 2 h after administration to ensure the motor-activity stimulating effects of LSD were no longer evident (see Rodriguiz et al., 2021). Subsequently, responses were assessed in all groups over post-injection days 1, 3, and 5. Immobility was higher in vehicle-treated VMAT2-HET than WT animals across days (Figure 6C)--indicating repeated testing was stable. In WTs, immobility times were similar following fluoxetine or 0.5 mg/kg lisuride over days. By contrast, 1 day after LSD and 1 mg/kg lisuride administration immobility in WTs increased relative to vehicle (*p*-values≤0.006). Additionally, immobility with LSD was higher than in fluoxetine and 0.5 mg/kg lisuride groups (*p*-values≤0.003). On days 3 and 5 no treatment effects were present. In VMAT2-HETs acute administration of fluoxetine, LSD, or 0.5 mg/kg lisuride reduced immobility times compared to vehicle (*p*-values≤0.043) (Figure 6C). Effects of 0.5 mg/kg lisuride and LSD were lost by day 1 post-injection, whereas immobility times for fluoxetine were lower than both lisuride doses (*p*-values<0.001) and those for LSD were decreased relative to 1 mg/kg lisuride (*p* = 0.014). By days 3 and 5, all treatment effects had vanished. Hence, 0.5 mg/kg LSD and lisuride were efficacious acutely, while fluoxetine reduced immobility in VMAT2-HETs up to 24 h after administration.

Anti-depressant like activities were evaluated also in the reserpinized mouse model as VMAT2 is a target for this drug (Erickson et al., 1996). C57BL/6 J mice received 1 mg/kg reserpine and after 3.5 h were given vehicle or 0.5 mg/kg lisuride and tested acutely in tail suspension 30-min later and on days 1, 3, and 5. Groups of VMAT2-HETs were used as comparisons. Immobility times for vehicle-vehicle C57BL/6 J mice were lowest, whereas those for reserpine-vehicle animals were among the highest across all test days (Supplementary Figure S6). Acutely, immobility in vehicle-vehicle controls was reduced relative to all groups (*p*-values ≤0.020) and immobility in 0.5 mg/kg lisuride VMAT2-HETs was lower than its vehicle (*p* = 0.042). Twenty-four hr post-injection (day 1) immobility was reduced in vehicle-vehicle *versus* reserpine-vehicle mice and in both VMAT2-HET groups (*p*-values ≤0.002). Additionally on day 1, immobility in reserpinized animals was higher than in reserpine plus lisuride mice and both VMAT2-HET groups (*p*-values ≤0.040). On days 3 and 5, immobility times were decreased in vehicle-vehicle mice compared to all other groups (*p*-values ≤0.005). In summary, immobility times in tail suspension were lowest in C57BL/6 J vehicle-vehicle controls across days, whereas 0.5 mg/kg lisuride showed anti-depressant like activities in VMAT2-HET and reserpinized C57BL/6 J mice, respectively, acutely and 24 h post-injection.

To determine if lisuride affected motor activities that could confound the tail suspension results, VMAT2 mice were tested in the open field. In both VMAT2 genotypes, 0.5 mg/kg lisuride depressed locomotor activities to similar extents *versus* vehicle (*p* < 0.001) (Supplementary Figure S7). A similar result has been reported with reserpine (Starr and Starr, 1994). Thus, the antidepressant drug-like actions of lisuride cannot be attributed to increased struggle activity in tail suspension.

In summary, the tail suspension and sucrose preference results reveal that 0.5 mg/kg lisuride possesses anti-depressant drug-like actions that last acutely or at least over 24 h.

## 3 Discussion

### 3.1 Motor performance

Locomotor, rearing, and stereotypical activities have been studied in various strains of rats—primarily males—and different strains of male and female mice. Lisuride most commonly produced a biphasic locomotor response where low doses decreased and higher doses were stimulatory (Carruba et al., 1980; 1985; Hara et al., 1982; Martin and Bendesky, 1984; Adams and Geyer, 1985; Fink and Morgenstern, 1985; Paulus and Geyer, 1991). Other researchers observed decreases (Carruba et al., 1985; Nisoli et al., 1992; Chen et al., 2023) or increases (Hlinak et al., 1983). In mice biphasic responses have been reported in the open field with lisuride (Martin and Bendesky, 1984). By contrast, in the behavioral pattern monitor (BPM) (Chen et al., 2023) locomotion was decreased and in our studies activities were reduced in both βArr strains with all lisuride doses in both genotypes of βArr mice. Thus, if βArr1 and βArr2 play any roles in the lisuride-induced suppression of locomotor activities in these mice, it is exceedingly minor.

In rats and mice, lisuride decreased rearing in the open field and BPM (Hlinak et al., 1983; Adams and Geyer, 1985; Chen et al., 2023) and our studies with βArr mice replicated these results. With respect to stereotypies, in rats lisuride elicited stereotyped sniffing (Hlinak et al., 1983; Carruba et al., 1985; Gerber et al., 1985), and licking or biting (Carruba et al., 1985). In the BPM with rats, hole-pokes were enhanced and the numbers of pokes within the same hole were augmented with increased lisuride doses (Adams and Geyer, 1985). In mice, licking, chewing, and gnawing were stimulated by lisuride (Horowski and Wachtel, 1976), while hole-poking in the BPM was suppressed (Chen et al., 2023). In our studies, both βArr strains displayed a biphasic U-shaped function to the different doses of lisuride.

### 3.2 Serotonin-syndrome like behaviors

To identify behaviors represented in stereotypy, responses were scored over the first 30 min post-injection interval. Behaviors associated with the 5-HT syndrome appeared in the βArr lines with the lowest lisuride dose and they consisted primarily of hunched posture, paw kneading, rigid posture, flat posture, Staub tail, and tremor or body shaking (see Habertzettl et al., 2013). Some of these behaviors had been observed previously in rats (Silbergeld and Hruska, 1979; Gerber et al., 1985; Marona-Lewicka et al., 2002). In our studies when cumulative responses were tabulated across doses, lisuride produced more of these responses in WT than in βArr1-KO mice but only with 4 mg/kg lisuride. Genotype differences were even greater in βArr2 mice, where the 0.1–4 mg/kg doses stimulated more 5-HT syndrome associated responses in WT than βArr2-KO animals. These results are potentially important for treating depression especially if the patient is currently taking tricyclic antidepressants or selective 5-HT reuptake inhibitors. In this case, anti-depressant treatments given in concert with psychedelics or, possibly with G protein HTR2A biased agonists, could facilitate expression of 5-HT syndrome in patients due to 5-HT toxicity (Haberzettl et al., 2013).

### 3.3 Other stereotyped behaviors

Psychedelics are reported to stimulate HTRs, grooming, and retrograde walking in rats and mice (Corne and Pickering, 1967; Kyzar et al., 2016; Rodriguiz et al., 2021), and HTRs and retrograde events are stereotyped and appear in 5-HT syndrome (Haberzettl et al., 2013). In rodents, HTRs have been taken as a proxy for hallucinogenic-like activities in humans (Corne and Pickering, 1967) and this substitution of behaviors is strengthened as deletion of *Htr2a* in mice abrogates this response (González-Maeso et al., 2007; Keiser et al., 2009). Moreover, the potency of psychedelics in the mouse HTR are correlated with the dose to elicit hallucinations in humans (Glennon et al., 1984; Halberstadt et al., 2020). In humans, lisuride has been reported to produce hallucinogenic activities in a single patient with prolactinoma (Turner et al., 1984), in a patient treated for migraine headache (Somerville and Herrmann, 1978), in five patients with Shy-Drager syndrome (Lees and Bannister, 1981), and occasionally in some patients with Parkinson’s disease (Schachter et al., 1979; Parkes et al., 1981; LeWitt et al., 1982; Vaamonde et al., 1991). However, in normal subjects lisuride is devoid of psychedelic effects up to 600 µg (Herrmann et al., 1977). In rats and mice, lisuride does not produce HTRs (Gerber et al., 1985; Halberstadt and Geyer, 2013) and in the present investigations, the numbers of HTRs were undifferentiated by genotype and they were excessively low in the βArr1 and βArr2 mice even up to the high 4 mg/kg dose.

With grooming, lisuride has been observed to suppress this response in rats (Ferrari et al., 1992). While no genotype effects were detected in our studies, lisuride decreased grooming in βArr1 mice but produced an inverted U-shaped response in βArr2 animals. The response differences between βArr strains in this and other tests may be due to different DNA libraries and strategies used to produce the *Arrb1* and *Arrb2* mutations, different embryonic stem (ES) cells used for DNA injection, different strains of mice used to implant the ES cells, and the mouse strains used for outcrossing the mutations (Bohn et al., 1999; Kim et al., 2018), Retrograde walking also was analyzed. In both βArr lines, lisuride produced a biphasic U-shaped dose-response curve. In summary, no genotype differences emerged for HTRs and retrograde walking, whereas grooming was reduced with lisuride in βArr1 animals while adopting an inverted U-shaped function in βArr2 mice.

### 3.4 Prepulse inhibition

Psychedelics disrupt PPI in rats (Sipes and Geyer, 1994; Johansson et al., 1995; Ouagazzal et al., 2001; Halberstadt and Geyer, 2010; Páleníček et al., 2010), mice (Rodriguiz et al., 2021), and humans (Vollenweider et al., 2007; Schmid et al., 2015). PPI is abnormal also in individuals with schizophrenia (Braff et al., 2001). Intriguingly, LSD-induced states are reported by some investigators to bear similarities to early acute phases of psychosis (Geyer and Vollenweider, 2008). In rats, lisuride was found to disrupt PPI (Halberstadt and Geyer, 2010). However, while the 5-HT2AR antagonist MDL 11,939 was unable to counteract lisuride’s effects, PPI was normalized with the D2/D3 antagonist raclopride (Halberstadt and Geyer, 2010). In the present studies, lisuride exerted no effect on PPI in βArr2 mice. By contrast, in βArr1 animals 0.5 mg/kg lisuride disrupted PPI. As with rats, the 5-HT2AR antagonist MDL100907 failed to rescue PPI in either βArr1 genotype. However, raclopride was efficacious in restoring PPI, but only in WTs; βArr1-KOs remained deficient. By comparison, the broad spectrum atypical antipsychotic clozapine, the D1 antagonist SCH23390, and the 5-HTR1B/D antagonist GR127935 normalized PPI in both βArr1 genotypes. Together, these results emphasize several points. First, it is surprising that βArr1 played a prominent role in the lisuride-induced PPI impairment through D2 receptors because much of the focus on this receptor in brain has been on βArr2 (Beaulieu et al., 2005; Allen et al., 2011; Urs et al., 2016). Second, since D1 and 5-HTR1B/D antagonists restored PPI in both βArr1 genotypes, these results suggest that G protein signaling through these receptors is critical to maintain sensory gating which could be important in certain psychiatric conditions.

### 3.5 Lisuride as an anti-depressant

Several small-scale clinical studies have indicated psilocybin and LSD can alleviate depression (Bonson and Murphy, 1996; Carhart-Harris et al., 2016; Griffiths et al., 2016; Ross et al., 2016; Reiff et al., 2020; McClure-Begley and Roth, 2022; Goodwin et al., 2023; Holze et al., 2023). Currently there are approximately 50 ongoing clinical trials with psilocybin, 2 trials with LSD, and additional trials with other psychedelics (see ClinicalTrials.gov). With respect to lisuride, there is evidence it may have anti-depressant actions in post-stroke patients experiencing depression (Hougaku et al., 1994). In C57BL/6 J mice, acute restraint-stress for 6 h is reported to produce increased immobility in forced swim and tail suspension with lisuride showing efficacy in both assays (Cao et al., 2022). However, these responses were tested acutely and immediately following a single session of restraint-stress. In the present investigation, a mouse genetic model of depressive-like behaviors was used to evaluate lisuride effects in sucrose preference and tail suspension (see Fukui et al., 2007). In the S-W pairing before lisuride treatment, WT mice had a strong preference for sucrose; VMAT2-HETs showed no preference. Hence, the mutants presented with anhedonia-like behavior. Following lisuride administration, sucrose preference in VMAT2-HET animals was robustly increased and remained high for 2 days, but declined thereafter. By contrast, preference was maintained in WTs across test-days. Thus, lisuride alleviated the anhedonia-like behavior in VMAT2-HET mice at least for 2 days, while sucrose preference in WTs was unaffected by lisuride.

In tail suspension, VMAT2 mice were treated with vehicle, fluoxetine, 0.3 mg/kg LSD, 0.5 or 1 mg/kg lisuride, and tested acutely and on days 1, 3, and 5 post-injection. Immobility times in vehicle-treated VMAT2-HETs were prolonged relative to WTs. In WT mice, immobility times were stable over days with fluoxetine and 0.5 mg/kg lisuride, while 1 mg/kg lisuride and LSD increased immobility acutely and on day 1. By comparison, in VMAT2-HETs administration of 0.5 mg/kg lisuride or LSD were efficacious acutely, while fluoxetine’s effects persisted through the next day. One mg/kg lisuride was ineffective. A limitation to our studies with VMAT2-HET mice lies in ascribing the specificity of lisuride’s anti-depressant actions to the 5-HTR2A. Unfortunately, the 5-HTR2A antagonist MDL100907 has anti-depressant-like activity in VMAT2-HETs (Kaplan et al., 2022) and its use to block lisuride’s effect would confound our experiment.

Since the VMAT2 is a target for reserpine (Erickson et al., 1996) and because patients treated with this drug frequently developed depression (Freis, 1954), we tested if reserpine treatment could mimic responses of VMAT2-HET mice. Reserpine produced high immobility in tail suspension with C57BL/6 J mice over all test days. Lisuride reduced the immobility in these reserpinized mice, but only 24 h after administration. By comparison, immobility in VMAT2-HETs was reduced acutely with lisuride.

To determine whether the lisuride-induced struggle activity in tail suspension was due to a lisuride stimulation of motor activity, mice were tested in the open field. Here, locomotion was reduced with 0.5 mg/kg lisuride in βArr1, βArr2, and in WT and VMAT2-HET mice—indicating lisuride effects on tail suspension were not coincident with this drug’s effects on motor activity. Collectively, lisuride relieved behavioral despair acutely and the anhedonia-like behavior at least for 24 h in VMAT2-HET mice. Despite these results, while parkinsonian patients are administered lisuride at least daily (Schachter et al., 1979; Parkes et al., 1981; LeWitt et al., 1982; Vaamonde et al., 1991), we could find no published reports of its efficacy in treating comorbid depression. Assuming bioavailability, pharmacokinetics, and other parameters are comparable between mice and humans, our experiments with VMAT2 mice suggest the efficacy of lisuride’s anti-depressant effects will not be maintained beyond 24 h in patients. Thus, lisuride as an antidepressant treatment would require at least daily administration to be effective.

### 3.6 Biased signaling and behavior

Lisuride and LSD bind with high affinities to 5-HT2ARs (Hoyer et al., 1994) and both ligands are partial 5-HT2AR agonists (Egan et al., 1998; Kurrasch-Orbaugh et al., 2003; Zhang et al., 2022). Despite similarities, LSD is hallucinogenic (Greiner et al., 1958), while lisuride is typically devoid of these effects in normal subjects (Herrmann et al., 1977). An interesting feature of LSD is its long-lasting psychedelic effects. This result may be attributed to the ability of the 5-HT2AR to “trap” LSD by forming a “lid” over the binding pocket with LSD and, thereby, slow the dissociation rate of this ligand from the receptor (Wacker et al., 2017; Kim et al., 2020). A similar result has been observed with lisuride (personal communication with Dr. Bryan Roth); however, its anti-depressant-like effects in mice appear transient. Thus, the differences between duration of LSD’s and lisuride’s effects may be due to their somewhat different receptor targets.

Recently, the receptor structures of the 5-HT2 family have been reported with LSD, psilocin, and lisuride (Gumpper et al., 1922; Kim et al., 2020; Cao et al., 2022). Both ergolines and psilocin bind at the bottom of the orthosteric binding-pocket of receptors in similar manners. However, these ligands bind also within the extended binding-pocket in subtly different ways that can produce slightly different receptor poses. For instance, the differential ergoline engagement with residue Y370 in the extended binding-pocket may affect transmembrane 7 positioning, thereby modulating βArr-mediated-signaling through the 5-HT2AR. Besides βArr, LSD activates G_q_, G_11_, and G_15_ proteins, with some activity at G_z_, and minimal activities at G_i_, G_12/13_, and G_s_ proteins (Kim et al., 2020). Similarly, lisuride stimulates G_q/11_ activation with calcium release (Cussac et al., 2008). Both βArr1 and βArr2 can be recruited to the 5-HT2AR *in vitro* (Bhatnagar et al., 2001) and βArrs are complexed with the receptor in cortical neurons *in vivo* (Gelber et al., 1999). Recently, it has been demonstrated that LSD is βArr biased at the 5-HT2AR *in vitro* (Wacker et al., 2013; Wang et al., 2013; Kim et al., 2020) and βArr2 biased *in vivo* (Rodriguiz et al., 2021). By comparison, our studies with βArr1-KO and βArr2-KO mice indicate lisuride is G protein biased at the 5-HT2AR since deletion of *Arrb1* or *Arrb2* results in very low incidences of HTRs coincident with WTs--even with the high 4 mg/kg dose. By comparison, LSD robustly stimulates HTRs in βArr1-KO and WT animals; however, this effect is lost in βArr2-KOs (Rodriguiz et al., 2021). Despite the lack of a differential effect of lisuride in βArr1-KO and βArr2-KO mice *versus* their respective WT controls, both βArr1 and βArr2 are expressed ubiquitously, with few exceptions, throughout the adult rodent brain (Gurevich et al., 2002). βArrs are present also within some of the same cells (Arriza et al., 1992; Bychkov et al., 2012). Importantly, both transfected βArr1 and βArr2 are recruited to HEK-293 transfected rat 5-HTR2A cells (Bhatnagar et al., 2001) and the 5-HTR2A is reported to be complexed with these βArrs in cortical neurons *in vivo* (Gelber et al., 1999). Hence, while our studies indicate lisuride is G protein biased, the loss of one βArr in given cells could have been compensated by the other βArr in our studies.

Since LSD exerts anti-depressant actions in humans (Gasser et al., 2015), we tested whether LSD, a 5-HTR2A βArr2 biased ligand (Wacker et al., 2017; Kim et al., 2020; Rodriguiz et al., 2021), and lisuride, a 5-HTR2A G-protein biased ligand, would have anti-depressant drug-like actions. These actions were confirmed in VMAT2-HET mice; however, they were short-lived in both cases. A possible limitation of our experiments is that LSD and lisuride bind many dopaminergic, adrenergic, and serotonergic receptors (Piercey et al., 1996; Egan et al., 1998; Marona-Lewicka et al., 2002; Millan et al., 2002; Kroeze et al., 2015). Hence, lisuride’s effects could be attributed to actions at other receptors or at their combinations of actions as in our present PPI published studies (Rodriguiz et al., 2021). A recent publication addresses this conundrum where a novel G protein biased 5-HT2AR compound was tested in VMAT2 and learned helplessness models with sucrose preference and/or tail suspension (Kaplan et al., 2022). The biased compound stimulated very low HTRs and was not reinforcing in conditioned place preference. Importantly, in VMAT2-HETs it showed anti-depressant drug-like actions in tail suspension that lasted for 2 days--the maximum tested. By comparison, in learned helplessness a single administration of the G protein biased compound lasted at least 3 days in sucrose preference--similar to psilocin. Immobility in tail suspension was reduced with the compound for 14 days, while psilocin was efficacious for 9 days. Results from this and the present investigation with lisuride suggest that G protein biased signaling at the 5-HT2AR may represent a novel way to separate the hallucinogenic-like potential of psychedelics from their anti-depressant drug-like actions.

## 4 Materials and methods

### 4.1 Animals

Adult male and female 3–8 month-old WT and global βArr1-KO (Kim et al., 2018), WT and global βArr2-KO (Bohn et al., 1999), WT and VMAT2-HET mice (Wang et al., 1997; Fukui et al., 2007), and C57BL/6 J mice (#000664; Jackson Laboratories, Bar Harbor, ME) served as subjects. All mouse strains had been backcrossed onto a C57BL/6 J genetic background for multiple generations. Mice were housed 3-5 per cage according to sex and genotype on a 12:12 h light:dark cycle (lights on 0700 h) in a humidity- and temperature-controlled room, with food and water provided *ad libitum*. Behavioral experiments were conducted between 0900–1700 h. All experiments were conducted with a protocol approved by the Institutional Animal Care and Use Committee at Duke University and according to ARRIVE guidelines.

### 4.2 Drugs

The drugs consisted of lisuride maleate (#4052), clozapine (#0444), SCH23390 (#0925), GR127935 (#1477; Bio-Techne Corporation, Minneapolis, MN), LSD-tartrate (NIDA Drug Supply Program, Bethesda, MD), fluoxetine HCl (#F132) and reserpine (#R0875; Sigma-Aldrich, St. Louis, MO). The vehicle for lisuride, LSD, MDL, reserpine, SCH23390, and GR127935 consisted of *N,N*-dimethyllacetamide (final volume 0.5%; Sigma-Aldrich) and it was brought to volume with 5% 2-hydroxypropoyl-β-cyclodextrin (Sigma-Aldrich) in sterile water (Mediatech Inc., Manassas, VA). Clozapine was solubilized with glacial acetic acid (0.05% final) and was brought to volume with 5% 2-hydroxypropyl-β-cyclodextrin in sterile water. Fluoxetine was solubilized in sterile water. All drugs were injected (i.p.) in a 5 ml/kg volume.

### 4.3 Open field activity

The open field consisted of a clear Plexiglas arena 21 × 21 × 30 cm (Omnitech Electronics, Columbus, OH) illuminated at 180 lux (Rodriguiz et al., 2021). Overhead cameras recorded behaviors using Media Player software (Noldus Information Technologies, Leesburg, VA). Mice were placed into the open field, 30 min later they were injected with the vehicle or different doses of lisuride, and immediately returned to the open field for 90 min. Fusion Integra software (Omnitech) recorded locomotor activity (distance traveled), rearing (vertical beam-breaks), and stereotypical activities (repetitive beam-breaks less than 1 s) in 5-min blocks.

### 4.4 Head twitch, grooming, retrograde walking, and serotonin syndrome-associated behaviors

These behaviors were filmed during the test for motor activity (Rodriguiz et al., 2021). All responses were scored over the first 30 min following administration of vehicle, 0.3 mg/kg LSD, or various doses of lisuride. HTRs and 5-HT syndrome-associated behaviors were scored by observers blinded to sex, genotype, and treatments using Observer XT 16 software (Noldus Information Technologies). Grooming and retrograde walking were scored by TopScan (Clever Sys Incorporated, Reston, VA). Behaviors that are analogues of 5-HT syndrome in rodents (see Haberzettl et al., 2013) included hunched postures, rigid postures while remaining standing, flat postures with limbs spread from the trunk, repetitive fore-paw kneading, Straub tail, and tremor, The data are presented as numbers of head twitches, time spent grooming, and numbers of retrograde walking and 5-HT syndrome-associated events.

### 4.5 Prepulse inhibition (PPI)

PPI of the acoustic startle response was monitored in SR-LAB chambers (San Diego Instruments, San Diego, CA) (Rodriguiz et al., 2021). Mice were administered vehicle or different doses of lisuride (dose-response studies) and returned to their home cages. For antagonist experiments, the vehicle, MDL100907, raclopride, SCH223390, or GR127935 were given 15 min prior to injection with vehicle or lisuride. Clozapine was administered 10 min before vehicle or lisuride. Following drug administration, the animals were placed into the PPI apparatus. After 10 min of habituation to a white-noise background (64 dB) a series of 42 trials was given. Trials were composed of 6 null, 18 pulse-alone, or 18 prepulse-pulse trials. Null trials consisted of white-noise, pulse trials were composed of 40 msec bursts of 120 dB white-noise, and prepulse-pulse trials consisted of 20 msec prepulse stimuli that were 4, 8, or 12 dB above the white-noise background (6 trials/dB), followed 80 msec later with the 120 dB pulse stimulus. Testing commenced with 10 pulse-alone trials followed by combinations of the prepulse-pulse and null trials, and was terminated with 10 pulse-alone trials. PPI responses were calculated as %PPI = [1–(pre-pulse trial amplitude/startle-only trial amplitude)]*100.

### 4.6 Sucrose preference

VMAT2 mice were housed individually for 7 days prior to and throughout the study (Fukui et al., 2007; Kaplan et al., 2022). Food was available *ad libitum*, while the water bottle was removed 2.5 h from the home-cage before the beginning of the dark cycle. A pair of metering bottles (Animal Care Systems, Centennial, CO) was filled with RO/Milli-Q water and the procedure was repeated until water consumption was stable and no side-bias evident. Subsequently, each pair of bottles was filled with water (W-W) or 0.5% sucrose (S-W). Note, the solution was prepared just prior to testing, the bottles were weighed before and immediately after testing, and they were cleaned and drained thoroughly each day after testing. The pair of bottles was presented to mice 1.5 h into their dark cycle (4 h total fluid deprivation) and they were allowed to drink for 2 h. Subsequently, bottles were removed and the original water bottle was returned to the home-cage at least 1 h later. The final W-W pairing occurred on day 1. The next day, animals were presented with the S-W pairing. On day 3 they were administered 1 mg/kg lisuride (i.p.) and 5 min later were given S-W. Subsequent S-W pairings were presented over days 4–6. The total volume of liquid consumed in 2 h was determined each day. Sucrose preference was calculated by dividing the volume of sucrose consumed by the total volume of liquid consumption.

### 4.7 Tail suspension

VMAT2 mice were administered vehicle, 20 mg/kg fluoxetine, or 0.5 or 1 mg/kg lisuride and tested 30 min later (see Fukui et al., 2007). When mice were given 0.5 mg/kg LSD, they were tested 2 h later since LSD significantly increases motor activity that lasts <2 h (Rodriguiz et al., 2021) that could confound this test. When reserpine was used, mice were administered a 1 mg/kg dose and 3.5 h later were given vehicle or 0.5 mg/kg lisuride and tested 30 min and 1, 3, or 5 days later. Mice were suspended by the tail for 6 min using medical tape attached to a Med Associates (St. Albans, VT) tail suspension hook connected to a load cell. The signal from the load cell processed with Med-Associates Tail Suspension Software that determined immobility times over the test session. All load cells were calibrated prior to testing each day according to the manufacturer’s instructions.

### 4.8 Statistics

Results are presented as means ±SEMs; all statistics were performed with SPSS 27 and 28 programs (IBM-SPSS, Chicago, IL). Univariate ANOVAs analyzed treatment effects. If cumulative baseline motor activities were significantly different for genotypes or treatment assignments, the cumulative post-administration results were analyzed by analysis of covariance. Repeated measures ANOVA were used for analysis of open field, PPI, sucrose preference, and tail suspension studies and, if significant, were followed by Bonferroni-corrected pair-wise comparisons. A *p* < 0.05 was considered statistically significant. The results displayed in all figures were plotted using GraphPad Prism 10.0.2 (GraphPad Software, Boston, MA).

## Data Availability

The original contributions presented in the study are included in the article/Supplementary Materials, further inquiries can be directed to the corresponding author.
