# Supplementary figures and images for: The G protein biased serotonin 5-HT2A receptor agonist lisuride exerts anti-depressant drug-like activities in mice
Source: Front Mol Biosci. 2023 Oct 10;10:1233743. doi: 10.3389/fmolb.2023.1233743 (PMC10603247; doi:10.3389/fmolb.2023.1233743)

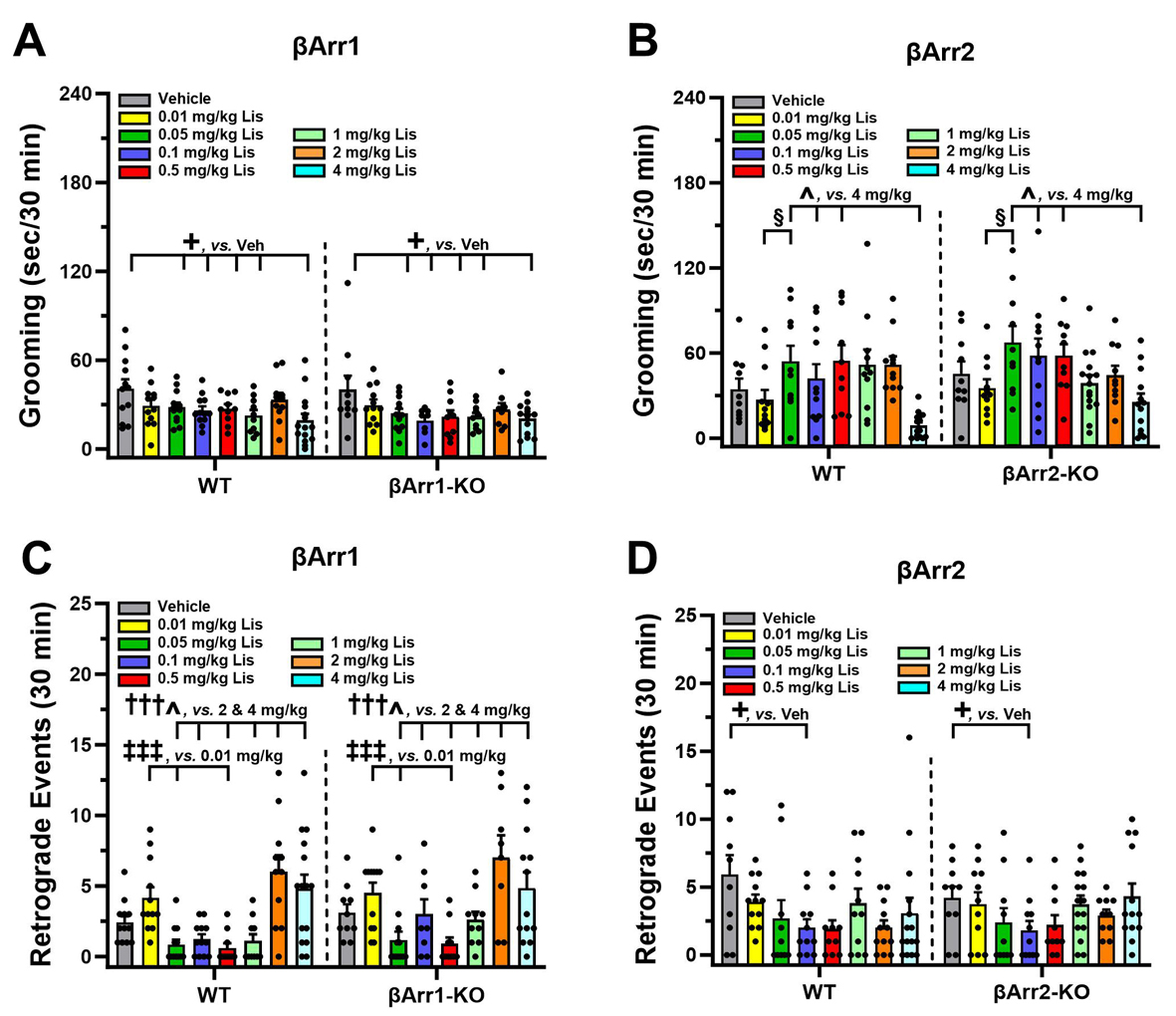

Supplement: Supplementary file 1 [file Image3.JPEG]

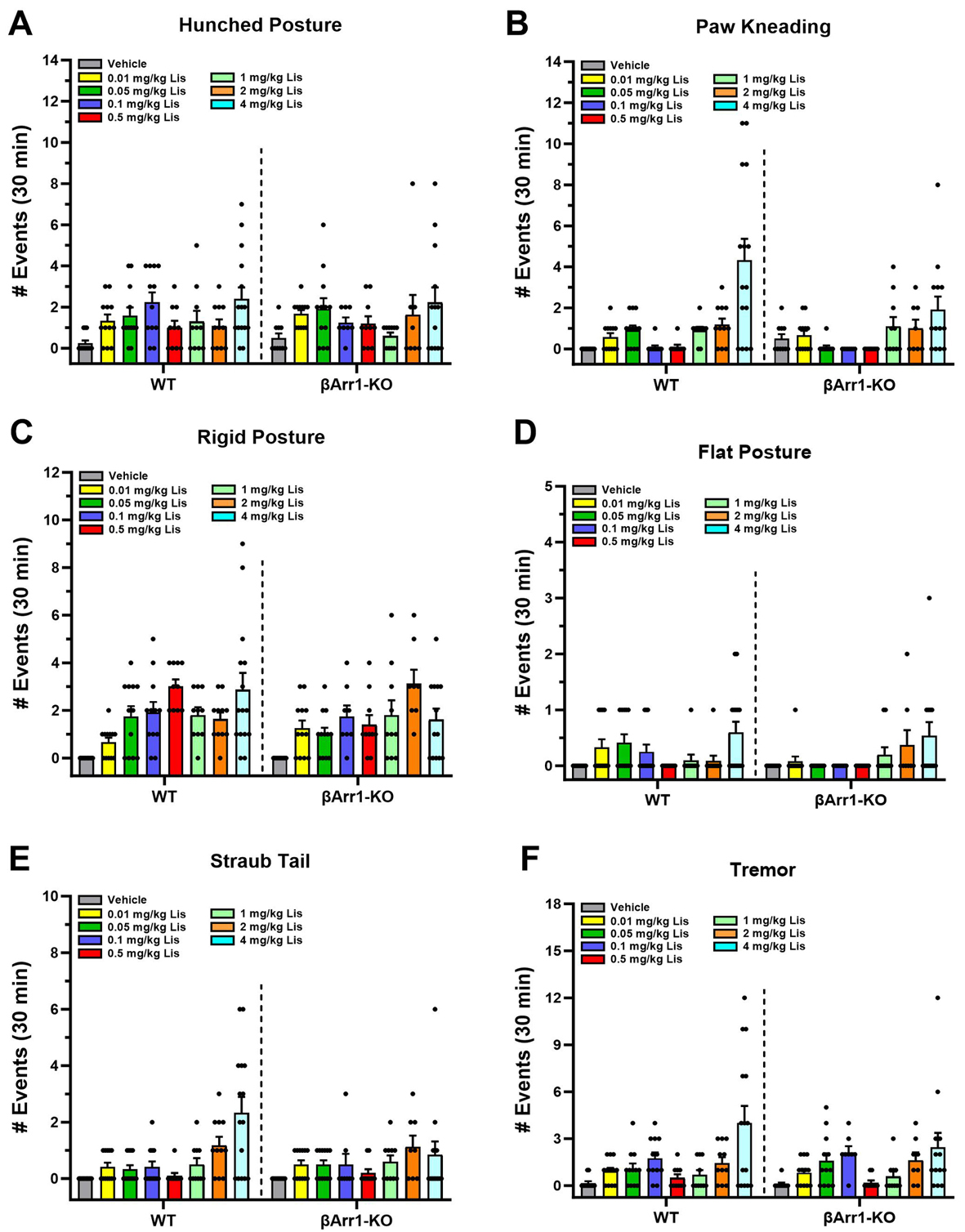

Supplement: Supplementary file 2 [file Image1.JPEG]

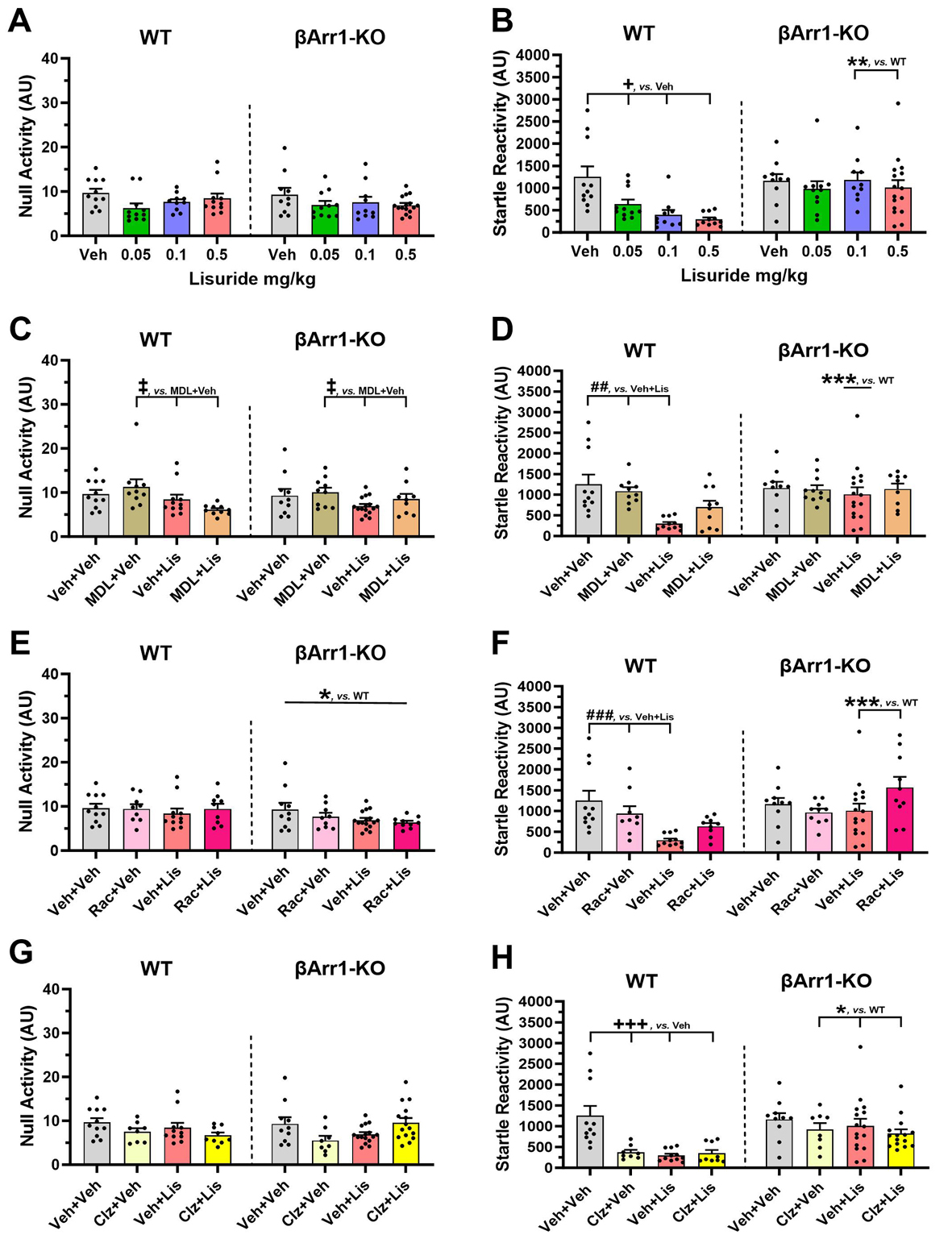

Supplement: Supplementary file 3 [file Image4.JPEG]

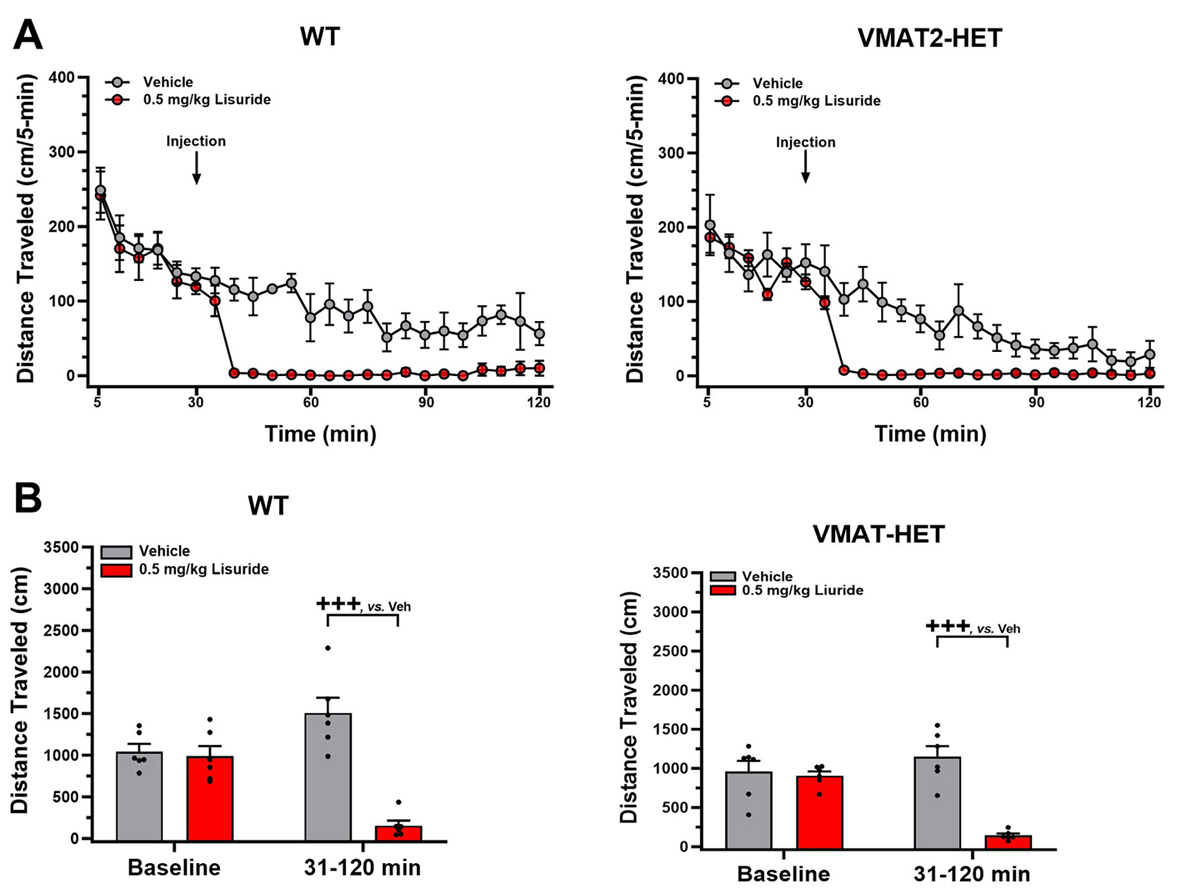

Supplement: Supplementary file 4 [file Image7.JPEG]

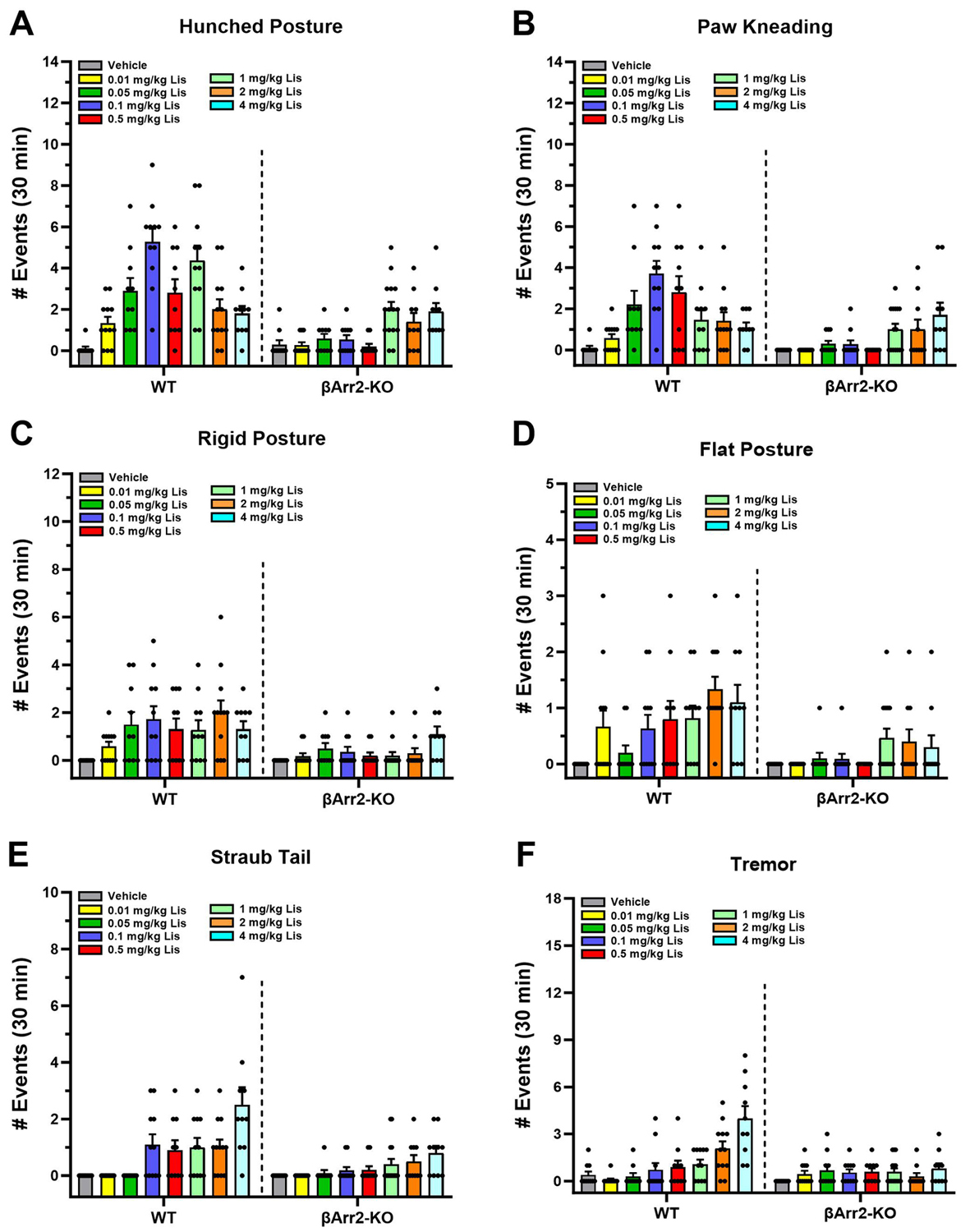

Supplement: Supplementary file 5 [file Image2.JPEG]

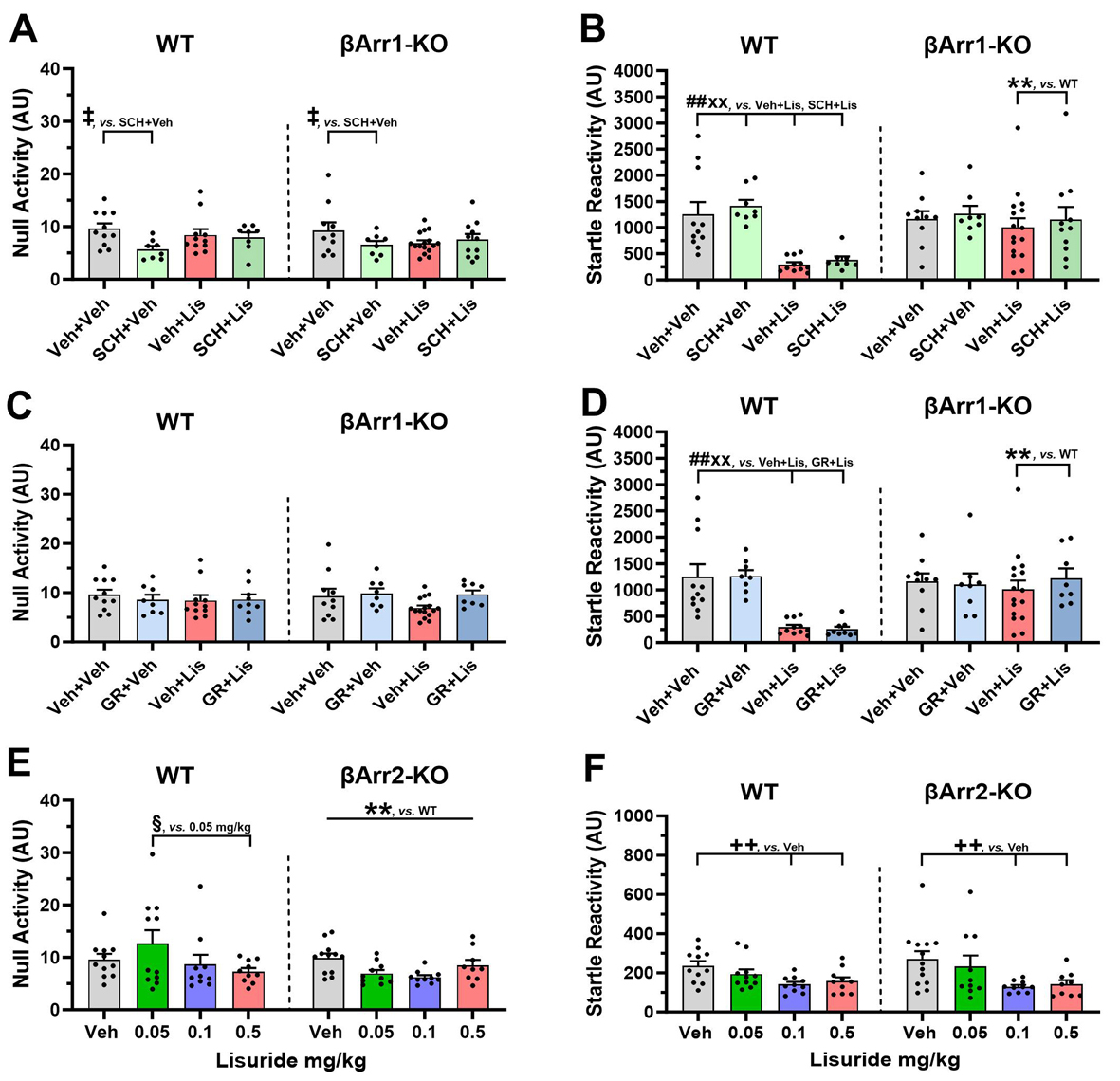

Supplement: Supplementary file 6 [file Image5.JPEG]

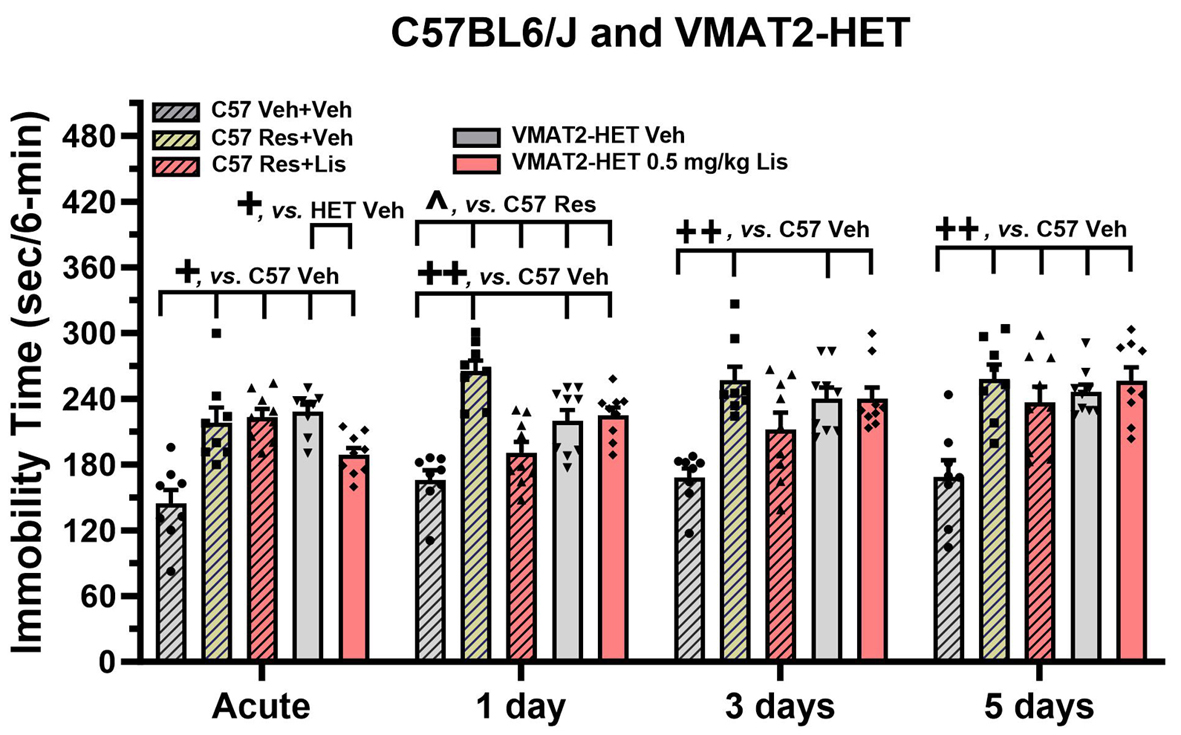

Supplement: Supplementary file 7 [file Image6.JPEG]
